# Views of patients with multi-morbidity on what is important for patient-centered care in the primary care setting

**DOI:** 10.1186/s12875-020-01144-7

**Published:** 2020-04-26

**Authors:** Sanne Jannick Kuipers, Anna Petra Nieboer, Jane Murray Cramm

**Affiliations:** Erasmus School of Health Policy & Management, Erasmus University Rotterdam, Rotterdam, The Netherlands

**Keywords:** Patient-centered care, Multi-morbidity, Primary care, Care delivery, Q-methodology

## Abstract

**Background:**

Patient-centered care (PCC) has been proposed as the way forward in improving primary care for patients with multi-morbidity. However, it is not clear what PCC exactly looks like in practice for patients with multi-morbidity. A better understanding of multi-morbid patients’ views on what PCC should look like and which elements are most important may help to improve care delivery for this vulnerable population. The present study thus aimed to identify views of patients with multi-morbidity on the relative importance of PCC aspects in a Dutch primary care setting.

**Methods:**

Interviews were conducted with 16 patients with multi-morbidity using Q-methodology, which combines quantitative and qualitative analyses. The participants ranked 28 statements about the eight dimensions of PCC (patients’ preferences, information and education, access to care, emotional support, family and friends, continuity and transition, physical comfort, and coordination of care) by relative importance. By-person factor analysis using centroid factor extraction and varimax rotation were used to reveal factors that represent viewpoints. Qualitative interview data were used to interpret the viewpoints.

**Results:**

The analyses revealed three factors representing three distinct viewpoints of patients with multi-morbidity on what is important for patient-centered care in the primary care setting. Patients with viewpoint 1 are *prepared proactive patients* who seem to be well-off and want to be in charge of their own care. To do so, they seek medical information and prefer to be supported by a strongly coordinated multidisciplinary team of healthcare professionals. Patients with viewpoint 2 are *everyday patients* who visit GPs and require well-coordinated, respectful, and supportive care. Patients with viewpoint 3 are *vulnerable patients* who are less resourceful in terms of communication skills and finances, and thus require accessible care and professionals taking the lead while treating them with dignity and respect.

**Conclusion:**

The findings of this study suggest that not all patients with multi-morbidity require the same type of care delivery, and that not all aspects of PCC delivery are equally important to all patients.

## Background

Increasing numbers of people face the burden of multi-morbidity [1, 2]. We define multi-morbidity as the co-existence of two or more chronic conditions in one patient. Patients with multi-morbidity are often considered to constitute a vulnerable and complex population with a high risk of mortality and high utilization of care, and they often are less satisfied with their care [3, 4]. Moreover, a systematic review showed that quality of life decreases with an increasing number of diseases [5]. In the Netherlands, general practitioners (GPs) coordinate care for patients with multi-morbidity [6]. However, the organization of high-quality primary care for this patient population is currently a great challenge in healthcare delivery. Primary care falls short of adequate and optimal care delivery for these patients, for whom single disease–oriented guidelines are not the most suitable [7, 8]. According to GPs, care delivery for this patient population is complex and demanding: There is a high medical complexity, clinical uncertainty on what is the best treatment, lack of communication between health and social care providers, and it is hard to always reach agreement on patient preferences regarding treatment goals [9]. This represents a missed opportunity, as the primary care setting is precisely the context identified as being most appropriate for effective management of patients with multi-morbidity [10].

Patient-centered care (PCC) has been proposed to be the way forward in improving primary care for patients with multi-morbidity [11]. The aims of PCC are to put patients at the center of their healthcare and to let them be in charge. PCC is associated with higher levels of social and physical well-being, and satisfaction with care, among patients with multi-morbidity [11]. The Picker Institute proposed eight dimensions of PCC [12]. First, the *patients’ preferences* dimension entails the treatment of patients with dignity and respect, taking their preferences into account and stimulating patients to set and achieve their own treatment goals. As PCC prioritizes placing patients in charge of their own care, the *information and education* dimension is also important to assure that patients should be well informed about all aspects of their care, regardless of their educational and migration backgrounds, or potential language barriers. Furthermore, patients must have good *access to care*, for example through easily made appointments, short wait times before consultations and accessible buildings. *Physical comfort* is also part of PCC because it is important to reduce potential feelings of pain, fatigue, shortness of breath, and lack of sleep. Other important aspects of physical comfort in GP practices are ensuring privacy, availability of comfortable chairs, and clean (waiting) rooms. PCC also entails *emotional support* since living with multiple chronic conditions is often accompanied by anxiety and depression [13, 14], and impacts patients’ private lives, such as their social relations or their jobs. However, chronic conditions often impact not only patients, but also their *family and friends*. PCC takes relatives into account, addressing their needs and questions, and involves the provision of adequate support to involve family members and friends in the care process. The *continuity and transition* dimension of PCC is important because multiple healthcare providers are often involved in care for patients with multi-morbidity. Information must be transferred adequately and referred patients must be well informed about where to go and why. Finally, to ensure the *coordination of care* among healthcare professionals within an organization (in this case, a GP practice) frequent deliberation in multidisciplinary team meetings is important, and patients should know who is coordinating their care and/or have a first point of contact [15]. Organizations with higher scores on these eight dimensions also report better organizational and patient outcomes [16, 17]. Although many organizations claim to be patient-centered, in reality this is not always the case. PCC delivery is often found to be more difficult for certain patient populations, among others low educated patients and ethnic minorities while these patients are precisely the ones who could really benefit from PCC [18–20].

Despite the thorough scientific description of PCC, it is still not clear what PCC looks like in practice for patients with multi-morbidity. The views and experiences of such patients are needed to identify the elements of PCC and its delivery that are most important to them, which may help to improve care delivery for this vulnerable population. Bayliss and colleagues have examined how patients with multi-morbidity describe ideal processes of care, that indeed entail patient-centeredness and individualized ways of care delivery; among others continuity in relationships with healthcare providers, clear communication, and accessible care [21]. However, patients with multi-morbidity are often described as one patient population, but as in single disease patients there are also differences among patients with multi-morbidity. Rijken and van der Heide (2019) found three subgroups of Dutch patients with multi-morbidity based on their background characteristics, medical characteristics and resources. This variety of patients with multi-morbidity requires different needs and ways of care delivery [22]. Thus, this is the first study to examine viewpoints of patients with multi-morbidity on the relative importance of PCC delivery-related aspects in a Dutch primary care setting.

## Methods

### Setting: the role of GPs in the Netherlands

Primary care systems in Europe vary widely, with different impacts on healthcare delivery design. The Netherlands has a strong primary care system based on a professional hierarchical gatekeeper model [23]. GPs have a central role in primary care, although a wide variety of care providers (e.g., dentists, pharmacists, dieticians, physiotherapists, and psychologists) are also involved. GPs function as gatekeepers, such that hospital and specialist care is often inaccessible without GP referral. Dutch GPs are often readily accessible [6, 23]; appointments can usually be made within two working days, and most GP-delivered care is covered by healthcare insurers (i.e., at no cost to patients). A standard consultation lasts 10 min [24]. For chronic conditions, however, often double consultations are scheduled. Each citizen is obligated to have basic health insurance (covering GP services), which can be complemented (voluntarily) by extra services, such as physiotherapy and/or dentistry [25]. Dutch GPs are in most cases non-interventionist; they handle 93% of all problems within primary care, only 4% of patients is referred to secondary care [6]. Most Dutch GPs are self-employed [23]. They coordinate mental healthcare (e.g. emotional support) as well as care for chronic diseases (e.g., diabetes, asthma, chronic obstructive pulmonary disease (COPD)) [6]. Patients can choose their own GP [6]; so, patients are often treated by the same GP every time they visit the GP practice.

### Participants

This study is part of a larger evaluation study investigating PCC for patients with multi-morbidity in the primary care setting in Noord-Brabant, the Netherlands [26]. In this larger evaluation study, a mixed-methods design was used to compare primary care practices aiming to improve PCC (intervention practices) with those providing care as usual (control practices). Patients were eligible to participate in this study when they had two or more registered chronic conditions (i.e. asthma, diabetes, COPD, heart and vascular disease). Patients with multi-morbidity from intervention practices who filled in a questionnaire were asked if they were willing to take part in the current study. Those who were willing to participate were contacted by telephone; they were given an in-depth explanation of the study and appointments were made to participate. Of 30 respondents who were willing to participate, 9 patients were ineligible due to visibility impairment (*n* = 2), illness preventing participation (*n* = 2), dementia (*n* = 1), and the inability to schedule an appointment (*n* = 4). Thus, a total of 17 patients consented to participate in the study. After the exclusion of one additional patient who could not complete the study tasks because she could not understand the instructions and statements, data from 16 patients were included in the analyses. Data saturation was reached.

In addition, four meetings with all healthcare professionals and researchers involved in the larger evaluation study were hosted. During these meetings the healthcare professionals (GPs and nurse practitioners) could share experiences and learn from each other. Furthermore, during these meetings preliminary research results were shared and validated.

The medical ethics committee of the Erasmus Medical Centre, Rotterdam, the Netherlands, determined that the rules laid down in the Medical Research Involving Human Subjects Act did not apply to this study (protocol no. METC_2018_021). Written consent was obtained from all participants.

### Q methodology

In this exploratory qualitative study, we used Q methodology to identify the perspectives of patients with multi-morbidity on which aspects of PCC are important. This approach combines quantitative and qualitative methods to examine subjectivity [27]. It is used to explore respondents’ personal experiences, tastes, values, and beliefs [28]. Q methodology has been used in research on primary care services [29, 30] and PCC [31, 32]. A Q-methodology study entails the following three steps: (a) design of the Q-set, (b) administering the Q-sort, and (c) statistical analysis and factor interpretation.

### Q-set design

The perspectives of patients with multi-morbidity on the importance of PCC aspects are generated by the placement of statements according to their relative importance. These statements about a subject matter are often referred to as the Q-set. An important characteristic of a Q-set is that it should fully cover the subject; PCC. Therefore, the current Q-set was developed based on the 36-item patient-centered primary care instrument [15]. It is not necessary to base a Q-set on a valid instrument, but we made use of the instrument because it assesses the eight dimensions of PCC among patients with multi-morbidity, and thus fully covers PCC. The number of statements in a Q-set depends on the subject matter. However, having too many statements is often considered demanding for participants [27]. As patients with multi-morbidity are often considered to be vulnerable, we decided to minimize the Q-set and use only three or four statements per dimension to reduce the complexity of the Q-sort and to shorten the interview time; the final Q-set consisted of 28 statements on PCC. The research team decided which items were merged (because they covered similar topics) to preserve the full coverage of PCC. To ensure comprehensibility and applicability, the Q-set was tested in a pilot study with two participants, and neither participant mentioned the need to include additional statements nor did they mention unclarities. Thus, agreement was reached on a final set of 28 statements (Table 2).

### Administering the Q sort (procedure)

**Fig. 1:**
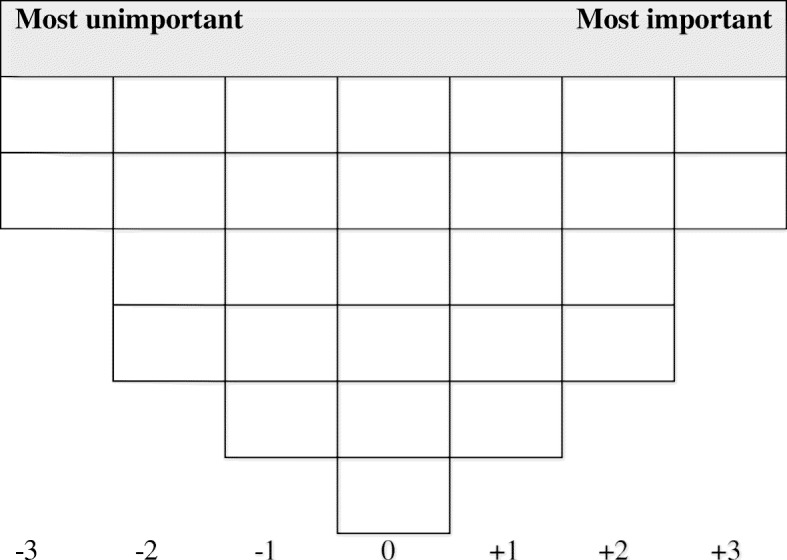
Q-grid

All interviews for the Q-study took place at the participants’ homes. The interviews lasted 45–90 min each and were conducted by the first author (SK). A script was used to ensure consistency. All interviews were recorded with participants’ permission. The participants were asked to rank the 28 statements according to their perceived importance for PCC in primary care. The statements were presented to the respondents on printed cards. After global instruction, the respondents were asked to read each of the statements and place it into one of three piles representing aspects of PCC that they consider to be “unimportant,” “neutral,” and “important.” The respondents were then asked to elaborate on their decisions. Then, the statements were sorted using a standardized Q-grid (Fig. 1) ranging from − 3 (least important) to + 3 (most important). First, respondents were asked to select the two statements that they considered to be most important from the “important” pile and to place them in the + 3 column. Second, the respondents chose the four statements that were most important from the remaining cards in the “important” pile and placed them in the + 2 column. This process was repeated for the “unimportant” pile, with the cards placed in the − 3 and − 2 columns. Lastly, cards from the “neutral” pile were placed in the remaining columns. When all cards are placed in the Q-grid, this is called a Q-sort. After completing the Q-sort, the respondents were asked to elaborate on their placement of statements in the four outer columns. All comments during the placements of the cards and the elaboration were transcribed verbatim.

### Statistical analysis

To make the methodology clear for all readers, this section is divided in three steps; 1. How to get factors out of Q-sorts, 2. Making factor arrays out of factors, and 3. How to interpret factor arrays. The PQ Method software was used to perform the statistical analysis [33].

#### How to get factors out of Q-sorts

All Q-sorts were manually imported in PQmethod. A correlation is used to simply measure the association or degree of (dis)agreement between the Q-sorts. All Q-sorts were intercorrelated. These correlations were subjected to a by-person factor analysis using centroid factor extraction and varimax factor rotation to reduce it to groups of participants who have ranked the statements in similar ways; these groups are also known as factors. The Kaiser–Guttman criterion was used to determine the number of factors extracted [34, 35]. A factor represents a viewpoint of patients with multi-morbidity about which aspects of PCC they consider to be important.

#### Making factor arrays out of factors

A factor thus represents a viewpoint, where some statements about PCC have higher loadings (e.g. a higher relative importance) compared to others. All Q-sorts that belong to a factor are merged by weighted averaging to form a so-called factor array; an ideal-typical Q-sort (see Table 2 for the factor arrays in this study).

#### How to interpret factor arrays

Thus, a factor array shows us which aspects of PCC are most important according to different viewpoints. It is this factor array that is the basis of different forms of factor interpretation. The aim of factor interpretation is to fully understand and explain the shared viewpoints. First, the patterning of items in the factor array was examined. Second, the comments and explanations that respondents gave during the Q-sort and follow-up interviews were used alongside. Specific attention was given to distinguishing statements (those placed in the + 3 and − 3 columns, e.g. where the viewpoints disagree). The interpretation of qualitative data helped to explain why a statement was important and to describe types of patients with similar perspectives.

## Results

Sixteen respondents participated in the study. Their sociodemographic characteristics are presented in Table 1. Their mean age was 72 years (range, 56–88 years). Nine participants were male and seven were female. Education levels ranged from primary school to university; three were low educated (primary school or less), 13 were high educated (ranging from secondary school to university). Nine participants were married, two were single, one was divorced, and four were widowed.

**Table 1:** Sociodemographic characteristics of study participants (*n* = 16)

Characteristic	Mean (range) or percentage
Age (years)	72.13 (56–88)
Gender (male)	56%
Education (low^a^)	18.8%
Marital status	
Single	12.5%
Married	56.25%
Divorced	6.25%
Widowed	25%

^a^primary education or less

The analyses revealed three factors that each represent a viewpoint. The factors explained 41% of the study variance. Data from 14 respondents were associated significantly with one of the three factors. Factors 1–3 were defined by data from six, five, and three respondents, respectively. Each factor is represented by a factor array, a composite Q-sort that represents the ideal/typical Q-sort, or shared perspectives/viewpoints. The factor arrays are shown in Table 2. Below, the three viewpoints are described with use of factor interpretation and follow-up interview data. In Q-methodology, each factor is given a name that captures the essence of the viewpoint (in this study 1) the prepared proactive patient, 2) the everyday patient, and 3) the vulnerable patient). Each viewpoint section starts with a detailed description supported by quotations, and ends with a brief summary of the viewpoint. Consensus statements, ranked similarly on all factors, are also provided.

**Table 2:** Statements and factor loadings

#		Factor 1	Factor 2	Factor 3
**Patient preferences**				
1	Being treated with dignity and respect	–1	3	3
2	Taking into account my wishes and preferences	–1	–1	0
3	Taking into account the influence that the treatment can have on my life	1	1	–1
4	Being supported to achieve my treatment goals	1	1	−2
**Physical comfort**				
5	Giving attention to my physical comfort (such as the management of pain, shortness of breath)	2	2	0
6	Clean and comfortable (waiting) rooms	–2	0	−1
7	Sufficient privacy in the treatment room(s) and at the counter	−1	0	0
**Coordination of care**				
8	That everyone is well informed; only having to tell my story once	0	−2	3
9	Well attuned care among the practitioners involved	2	2	0
10	A contact person who knows everything about my illness and care	3	− 3	0
11	Being able to easily contact someone with questions	1	2	1
**Continuity of care**				
12	Being well informed about where to go and why when referred to another care provider (specialist/dietician/physiotherapist)	2	0	2
13	With a referral, all my information is passed on correctly	1	0	1
14	Advice (such as on medication) from different practitioners (medical specialists and family doctor) is well attuned	2	2	1
**Emotional support**				
15	Emotional support	−3	1	−1
16	Paying attention to possible feelings of fear, gloom, and anxiety	−2	1	−2
17	Paying attention to the impact of my health on my private life (family, relatives, work, social life)	−3	−1	−2
**Access to care**				
18	Not having problems going from my home to my family doctor and back again	0	−2	2
19	Free, available care and medication (without extra payment)	−2	−1	2
20	Easily and quickly scheduling an appointment	0	1	2
21	Not having to wait long before it is my turn at an appointment	−1	−2	0
**Information and education**				
22	Being well informed	3	0	−1
23	A good explanation for all the information I receive	0	0	1
24	Easy access to my own data (lab results, medication overview, referrals)	1	−1	−1
25	Being able to ask all the questions I want	0	3	1
**Family and friends**				
26	Involving relatives in my treatment	−2	−2	−3
27	Giving attention to care and support provided by family members	0	−3	−2
28	Giving attention to possible questions or needs from my family members	−1	−1	−3

### Viewpoint 1: the prepared, proactive patient

This is the viewpoint of six patients, of whom four are male (67%), all high educated (100%), four married (67%), two widowed (33%), and the mean age of the patients holding this viewpoint is 73 years old.

Patients holding viewpoint 1 consider the information and education dimension of PCC to be important. To be in charge of their own care and well prepared for GP visits, they need to be informed about all aspects of their care (statement 22, + 3), and they want to be informed about where to go and why when they are referred to other care providers (statement 12, + 2).

“Very important, yes, of course. The family doctor has to find out where the distress is coming from, and whether I have to go to a lung specialist or a nephrologist. And when she refers me, I have to know why I have to go to that specific specialist.” (Respondent 1, statement 12)

These patients have a strong focus on care related to their physical comfort (statement 5, + 2). They consider their GPs’ main task to be the maintenance of their physical comfort, through pain management and the treatment of shortness of breath.

"I find that promoting my physical comfort is at the heart of what I can expect from a general practitioner." (Respondent 1, statement 5)

They consider, however, aspects of physical comfort that are not related to their physical health, such as the comfort of GP waiting rooms (statement 6, − 1), to be less important.

"Of course, the waiting room shouldn't be dirty, but it's not comfortable here. And I don't mind." (Respondent 1, statement 6)

Furthermore, these patients do not consider privacy (e.g., in GP waiting rooms) to be an important aspect of PCC. For instance, they do not mind when others hear them speak about their illnesses (statement 7, − 2).

"I don’t mind when everyone knows what's wrong with me." (Respondent 9, statement 7)

Patients holding this viewpoint prefer a well-coordinated multidisciplinary team of healthcare professionals with a central contact person who knows everything about their illness and care (statement 10, + 3), and they prefer care to be well attuned among the professionals involved (statement 9, + 2).

"I think that you should have someone who has insight into your health and care. That's probably because I haven't been so well informed myself for a number of years. But then at least they know what's going on. Yes, I think that's very important." (Respondent 2, statement 10)

These patients also seem to be financially well-off. They have no problem paying for costs not covered by their insurance when required to receive good care (statement 19, − 2).

"Sometimes you have to take medicines, but you have to pay extra. But you really need them, so that is not an issue. But I know I might be in different circumstances compared to others, because you have to be able to make it financially as well." (Respondent 16, statement 19)

Summary: Patients with this viewpoint like to be in charge, and will, when possible, contribute to their own care delivery. During GP visits, they are often well prepared and focus primarily on the medical aspects of their care, seeking (new) information about their conditions. These patients do not consider emotional support to be the responsibility of GPs; the main focus should be on patients’ physical health. They like to be supported by a well-coordinated multidisciplinary team of healthcare professionals, and they seem to be well-off and down-to-earth.

### Viewpoint 2: the everyday patient

This is the viewpoint of five patients, of whom two are male (40%), four high educated (80%), two married (20%), two single (20%), one widowed (20%), and the mean age of patients holding this viewpoint is 65 years old.

Patients holding viewpoint 2 highly value the patients’ preferences dimension of PCC. They want to be taken seriously and to establish good relationships with healthcare professionals (statement 1, + 3).

"That's very important. Because whether you are a millionaire or a farmer, you should be respected anyhow." (Respondent 11, statement 1)

According to these patients, the ability to ask any question (statement 25, + 3) is an important aspect of a trusting relationship with one’s healthcare professional (statement 25, + 3). They feel that barriers to open communication will negatively impact care delivery and, thus, the quality of care.

"If you go to a doctor with a certain threshold, so if I'm afraid to ask certain things, I don't think a doctor can treat me well. But if I come with a certain ailment and I don't show the back of my tongue about what I feel or what I think I feel, how should they act correctly? When I go to my doctor, I must indeed feel myself in such a relaxed way that I can and dare say anything. Even if they don't agree, or I don't agree with them, it has to be possible to talk with each other." (Respondent 14, statement 25)

In contrast to those holding viewpoint 1, these patients do not need a central contact person who knows everything about their care (statement 10, − 3). These two patient groups, however, interpreted this statement differently. Patients with viewpoint 2 consider a central contact person to be yet another care provider, whereas those with viewpoint 1 consider this person to be more of a case manager. Patients with viewpoint 2 want to handle all communication themselves, to speak for themselves and avoid misinterpretation.

"Why do I need a contact person who knows about my illness or treatment? I can say for myself what I want and what I don't want." (Respondent 11, statement 10)

"Another contact. The more contacts, the more things go wrong. Now I have two short lines; the nurse practitioner, the GP, and them together, who of course also communicate about me. I also know what is being communicated, which is important. If there is another contact, how will I be sure that they'll communicate it to the third party the way I want, or whether they correctly interpret my answers and my questions?" (Respondent 14, statement 10)

In accordance with those holding viewpoint 1, patients holding viewpoint 2 consider the PCC dimension of access to care to be less important. They do not mind waiting for their appointments because they value their GPs’ help and they grant other patients this valued time as well, even if that means longer wait times (statement 21, − 2).

"No, I don't mind. Occasionally you experience that someone needs more time. And I don't mind. Especially when it's urgent." (Respondent 4, statement 21)

In addition, they do not consider traveling to their GPs’ offices to be an important issue (statement 18, − 2).

"I can get there easily, because I have a part-time taxi pass. And otherwise I can take the bus, but then I have to walk a bit through the forest." (Respondent 6, statement 18)

Continuity of care is important for patients with this viewpoint. These patients do not mind cooperating with care providers to guarantee continuity of care; when needed, they do not mind telling their stories several times (statement 8, − 2).

"I don't think that's important, because I want to tell my story if necessary, to the right healthcare provider. I think that if you have something, you want to give an explanation at that moment and ask questions that fit in with that moment. You can read everything in the file, but that doesn't have to apply at that moment. This may also include things that you have processed and let go of again." (Respondent 15, statement 8)

Advice from the different healthcare providers involved in care for patients with multi-morbidity can be difficult to align. Such alignment, for example regarding medication (statement 14, + 2), is important for patients holding viewpoint 2 to ensure that they receive safe, high-quality care. They find contradictory advice to be counterproductive.

"The GP gave me advice, but the therapist gave contradictory advice. Another therapist gave the same advice as the GP. The advice [of the first therapist] was nonsense advice and the second therapist agreed, it would only be counterproductive." (Respondent 14, statement 14)

"Yes, I think that's very important. I also get medication sometimes. I don't take that much, only three. But I'm paying close attention to other packaging. Do they contain the same medicines that I had? That's what I asked the other day at the pharmacy. You get different boxes every time, but they explained that is because they are cheaper and they contain the same medicines that I must have. So that's important, because you don't know if all those other medicines will work the same way." (Respondent 6, statement 14)

Summary: Patients with this viewpoint represent the average patient who visits the GP. Similar to patients with viewpoint 1, these patients prefer to be supported by a well-coordinated multidisciplinary team. They seem to be less informed about their conditions than are patients holding viewpoint 1, and thus feel that the ability to easily turn to their healthcare professionals with all of their questions is important. In addition, they want to receive relevant medical information and advice concerning their conditions and care. Furthermore, they highly value trusting relationships with their healthcare professionals and want to be treated with dignity and respect.

### Viewpoint 3: the vulnerable patient

This is the viewpoint of three patients, of whom two are male (67%), two married (67%), one divorced (33%), one high educated (33%), and the mean age of patients holding this viewpoint is 79 years old.

Like those with viewpoint 2, patients holding viewpoint 3 value the patients’ preferences dimension of PCC, as they feel strongly that being taken seriously and being treated with dignity and respect by their healthcare professionals are important (statement 1, + 3).

"Yes, that's important. You are a human being. You just want to be treated normally." (Respondent 7, statement 1)

These patients also agree that access to care is very important; they greatly value being able to travel to their GPs’ offices without problems (statement 18, + 2) and being able to schedule appointments easily and within a reasonable timeframe (statement 20, + 2).

"Yes, I have a problem with that [traveling to the GP practice]. I can get there, but I have to leave my mobility scooter outside. Then I have to go upstairs with the elevator and then I have to walk a bit. And a bit in the waiting room and to the toilet as well. I can't do that." (Respondent 7, statement 18)

The affordability of care is also important to these patients, who seem to have fewer financial resources than do those holding viewpoint 1. For example, the need to pay costs not covered by insurance is an issue for patients holding viewpoint 3 (statement 19, + 2).

"Yes, altogether I have 70 euros a week. I have my General Old-Age Pensions Act money, but that's not much." (Respondent 7, statement 19)

Another patient agreed on this statement as well.

"Yes, I think that's important. The care is already so expensive, we already pay so much per month [for the health insurance]." (Respondent 8, statement 19)

These patients seem less capable of truly comprehending information, and experience more difficulties in communicating with healthcare professionals than do patients with viewpoints 1 and 2. Shared decision making with patients holding viewpoint 3 is thus more challenging. As a result, these patients do not want to set their own treatment goals; they would rather leave this task to their healthcare professionals (statement 4, − 2).

“The doctor determines what needs to be done. These doctors have an understanding of treatment goals.” (Respondent 12, statement 4)

“All those terms are so difficult. No, you just have to say; that's what you need. Not all those Latin words. I just want to be treated as I am.” (Respondent 7, statement 4)

Given their struggles with communicating, these patients are often asked to re-tell their stories, which they dislike (statement, 8, + 3).

“It would be nice if I didn't have to tell them every time. Every time I come, every time, I have to say; I have these medicines.” (Respondent 7, statement 8)

Summary: Patients with this viewpoint need more support regarding their care than do patients with viewpoints 1 and 2. These patients seem to be vulnerable in terms of communication skills and finances. They are aware of their lack of resources; they are looking for affordable and accessible care provided by healthcare professionals who take them seriously and treat them with dignity and respect. They are less focused on the way in which care is delivered than are patients with viewpoints 1 and 2; in their opinion, such matters fall under the expertise of their GPs and nursing practitioners.

### Consensus among viewpoints

Although the three patient viewpoints differ from each other, they contain agreement on some elements of PCC. All viewpoints consider three aspects of continuity of care to be important for PCC: being well informed about where to go and why when referred to other care providers (statement 12), accurate transfer of information upon referral (statement 13), and alignment of advice from different practitioners (statement 14). Patients also agree on the importance of two aspects of coordination of care: attunement of care among all practitioners involved (statement 9) and the ability to easily contact someone with questions (statement 11). Patients think that attention should be paid to the influences of treatments on their lives (statement 3), but do not need all of their wishes and preferences to be taken into account (statement 2). Finally, almost all patients classified good explanation of all relevant information (statement 23) as neutral.

Within all viewpoints, patients consider two PCC dimensions to be less important: emotional support, and family and friends. First, patients do not think that emotional support is a key task of GPs; they would rather seek such support elsewhere (statement 15, − 3/1/− 1). The impact of their health on their private lives (statement 17, − 3/− 1/− 2) and possible feelings of fear, gloom, and anxiety (statement 16, − 2/1/− 2) were less important than other aspects of PCC to all patients. Some patients do not seem to have given much thought to whether emotional problems should be discussed with their GPs, and some patients’ chronic diseases have not really affected them emotionally.

“I don't think it's that important. This [emotional support] has absolutely nothing to do with my illness.” (Respondent 12, statement 15)

Second, patients do not wish to involve their family members or friends in their care at present (statement 26, − 2/− 2/− 3) because they do not want to bother others, because they feel that their condition is a private matter, or because they simply feel that they can handle their care on their own. Thus, they also feel that attention to the needs and support provided by their relatives is not important (statement 27, 0/− 3/− 2); statement 28, − 1/− 1/− 3). However, they believe that involving family and friends could be beneficial in more severe stages of illness (e.g., cancer, terminal illness). During these stages, optimal provision of emotional support becomes a crucial aspect of PCC.

"I'll take care of it myself. I have severe COPD, but it's never been so severe that my family should be informed by my GP. Maybe if I ever get terminally ill, it would be important someday." (Respondent 14, statement 27)

## Discussion

This study aimed to explore the relative importance of PCC-related aspects in a primary care setting according to patients with multi-morbidity in Noord-Brabant, the Netherlands. Three viewpoints regarding these aspects were identified. Patients with viewpoint 1 are the *prepared proactive patients* who seem to be well-off and want to be in charge of their own care. To do so, they seek medical information and prefer to be supported by a strongly coordinated multidisciplinary team of healthcare professionals. Patients with viewpoint 2 are *everyday patients* who visit GPs, and are in need of well-coordinated, respectful, and supportive care. Patients with viewpoint 3 are *vulnerable patients* who are less resourceful in terms of communication skills and finances, and are thus in need of accessible care and professionals’ lead taking while treating them with dignity and respect.

The findings of this study suggest that not all patients with multi-morbidity are in need of the same type of care delivery, and that the PCC dimensions are not equally important to all patients. This is in accordance with a study by Rijken and van der Heide (2019) that identified subgroups of patients with multi-morbidity based on their care needs and support [22]. Rijken and van der Heide (2019) showed that subgroups of patients with multi-morbidity can be identified based on differences with regard to, among others, physical functioning, social functioning, mental health, and emotional functioning [22]. Interestingly, differences still existed after controlling for physical condition and age. Background characteristics, medical characteristics and resources were examined as well. Though we did not use an instrument to measure these aspects in this study, we did gather qualitative data on these characteristics. One of the groups in the study from Rijken and van der Heide is limited with regard to financial resources, and communicative skills [22], which seems to correspond with our group 3 ‘the vulnerable patient’. Our findings are also in accordance with those of previous research examining the influence of health literacy on primary care needs. Health literacy encompasses several resources conferring the capacity to meet the complex demands of healthcare management [36]; it is fundamental for patients who want to be in charge of their own care [37]. The needs of patients with low and high health literacy differ in relation to the ability to manage their own care, for example with regard to communication and information provision. Our results provide insight that can guide the design of PCC with adjustment according to the diversity of care needs of patients with multi-morbidity. We recommend further research to explore whether the adjustment of care according to these different viewpoints results in better patient outcomes.

Our results also showed that patients consider the dimensions ‘family and friends’ and ‘emotional support’ to be less important within all three viewpoints. Patients’ ranking of the involvement of family and friends as less important than other PCC dimensions may reflect their perceived disease severity. They indicated that this dimension may become more important in more severe stages of illness, including terminal illness. These results may be related to previous findings that patients with chronic illnesses involve their family members and friends more often when their care needs are complex and when they are more vulnerable to worse health outcomes [38, 39].

Many participants in our study did not consider emotional support to be a key GP task, although the 2014 reform of mental healthcare in the Netherlands designates it as such [6]. A possible explanation for this result is that patients are simply not used to this change in the GP role. Although patients do not expect emotional support from their GPs, they need such support in general. Coping with multiple chronic diseases is often accompanied by psychological burden, and patients with chronic conditions are at increased risk of developing depression and anxiety [40, 41]. This emotional burden necessitates good PCC.

This study has several limitations. First, our sample may be considered small. However, a large sample is not required for the application of Q-methodology, and our sample size is similar to those of other Q-studies [42, 43]. Data saturation and the representation of all viewpoints are more important than the sample size. We achieved data saturation (respondents gave no additional answers or explanations during final interviews), with the identification of three viewpoints. On presentation of the preliminary study results to all involved professionals (GPs and nurse practitioners), the professionals recognized the three viewpoints and agreed that they fully described this patient population; in their expert opinions, no viewpoint was missing. Therefore, the sample size in this study should not be considered problematic. It should be noted that since data saturation is somewhat subjective, further studies would be necessary to make sure no viewpoints are missing. Second, during interpretation of our findings it should be taken into account that our sample may still be biased, since those excluded from the study, or the non-responders from the larger evaluation study, may have been in poorer health compared to those who participated in the Q-study. We do not have health literacy scores or deprivation scores of the participants. Third, using Q-methodology forced us to exclude participants, because some were too ill to participate, and some visible impairments made it impossible for participants to read the cards and rank them according to their relative importance. Fourth, the generalizability of our results may be limited, as this study was conducted in Noord-Brabant, the Netherlands. Therefore, further research in other regions and countries with different primary care systems is needed to confirm and expand on our study findings. Since the Netherlands has a strong primary care system, it is possible that a replication of this study in a country with a different primary care system may result in different study findings. Moreover, Q-methodological findings do not allow for generalizability to an entire population what makes it more difficult to draw conclusions. However, this does not mean that findings based on Q-methodology cannot have wider implications [44]. Fifth, it should be considered that the qualitative part of this study has a risk of bias towards the researcher that conducted the interviews and selected the statements. We tried to minimize this bias by using a script for all interviews. However, and that is directly the strength of Q-methodology, both quantitative and qualitative analyses revealed similar results. Finally, the lesser communication skills of patients with viewpoint 3 impacted our findings, as these patients had greater difficulty elaborating on their Q-sorts and thus provided less-rich qualitative descriptions of their views than did patients with viewpoints 1 and 2. Patients with viewpoint 3 did, however, have strong opinions about which aspects of PCC they considered to be more and less important for their care, and had no problem Q-sorting the statements.

## Conclusion

Using Q-methodology, we identified three viewpoints held by patients with multi-morbidity on the important aspects of PCC delivery in the primary care setting, representing [1] the prepared proactive patient, [2] the everyday patient, and [3] the vulnerable patient. The results of this study are important for improving care delivery for patients with multi-morbidity in primary care. The findings of this study suggest that not all patients with multi-morbidity require the same type of care delivery, and that not all aspects of PCC delivery are equally important to all patients. This knowledge is important for healthcare professionals in the primary care setting to be able to tailor their care to the needs of patients with multi-morbidity to ensure the best possible outcomes for their patients. The results can make GPs more aware of the viewpoints on PCC-related aspects and provide more insight in what PCC may look like in practice for this specific patient population.

## Data Availability

The data are available upon reasonable request.

## Abbreviations

COPD: chronic obstructive pulmonary disease
GP: general practitioner
PCC: patient-centered care
